# Calorie restriction improves metabolic state independently of gut microbiome composition: a randomized dietary intervention trial

**DOI:** 10.1186/s13073-022-01030-0

**Published:** 2022-03-14

**Authors:** Solomon A. Sowah, Alessio Milanese, Ruth Schübel, Jakob Wirbel, Ece Kartal, Theron S. Johnson, Frank Hirche, Mirja Grafetstätter, Tobias Nonnenmacher, Romy Kirsten, Marina López-Nogueroles, Agustín Lahoz, Kathrin V. Schwarz, Jürgen G. Okun, Cornelia M. Ulrich, Johanna Nattenmüller, Arnold von Eckardstein, Daniel Müller, Gabriele I. Stangl, Rudolf Kaaks, Tilman Kühn, Georg Zeller

**Affiliations:** 1 German Cancer Research Center (DKFZ), Division of Cancer Epidemiology, Heidelberg, Germany; 2 Medical Faculty, Heidelberg University, Heidelberg, Germany; 3 European Molecular Biology Laboratory (EMBL), Structural and Computational Biology Unit, Heidelberg, Germany; 4 Molecular Medicine Partnership Unit, Heidelberg, Germany; 5 Institute of Agricultural and Nutritional Sciences, Martin Luther University Halle-Wittenberg, Halle (Saale), Germany; 6 Heidelberg University Hospital, Diagnostic and Interventional Radiology, Heidelberg, Germany; 7 Biobank of the National Center for Tumor Diseases (NCT) Heidelberg, Heidelberg, Germany; 8 Analytical Unit, Biomarkers and Precision Medicine Unit, Health Research Institute Hospital La Fe, Valencia, Spain; 9 Department of General Paediatrics, Division of Neuropediatrics and Metabolic Medicine, University Hospital Heidelberg, Dietmar-Hopp Metabolic Center, Heidelberg, Germany; 10 Huntsman Cancer Institute and Department of Population Health Sciences, University of Utah, Salt Lake City, UT USA; 11 Institute of Clinical Chemistry (IGFS), University Hospital Zurich, Zurich, Switzerland; 12 Institute for Global Food Security, Queen’s University Belfast, Belfast, Northern Ireland, UK; 13 Heidelberg Institute of Global Health (HIGH), Faculty of Medicine and University Hospital, Heidelberg, Germany

**Keywords:** Obesity, Overweight, Weight loss, Gut microbiome, Intermittent calorie restriction

## Abstract

**Background:**

The gut microbiota has been suggested to play a significant role in the development of overweight and obesity. However, the effects of calorie restriction on gut microbiota of overweight and obese adults, especially over longer durations, are largely unexplored.

**Methods:**

Here, we longitudinally analyzed the effects of intermittent calorie restriction (ICR) operationalized as the 5:2 diet versus continuous calorie restriction (CCR) on fecal microbiota of 147 overweight or obese adults in a 50-week parallel-arm randomized controlled trial, the HELENA Trial. The primary outcome of the trial was the differential effects of ICR versus CCR on gene expression in subcutaneous adipose tissue. Changes in the gut microbiome, which are the focus of this publication, were defined as exploratory endpoint of the trial. The trial comprised a 12-week intervention period, a 12-week maintenance period, and a final follow-up period of 26 weeks.

**Results:**

Both diets resulted in ~5% weight loss. However, except for *Lactobacillales* being enriched after ICR, post-intervention microbiome composition did not significantly differ between groups. Overall weight loss was associated with significant metabolic improvements, but not with changes in the gut microbiome. Nonetheless, the abundance of the *Dorea* genus at baseline was moderately predictive of subsequent weight loss (AUROC of 0.74 for distinguishing the highest versus lowest weight loss quartiles). Despite the lack of consistent intervention effects on microbiome composition, significant study group-independent co-variation between gut bacterial families and metabolic biomarkers, anthropometric measures, and dietary composition was detectable. Our analysis in particular revealed associations between insulin sensitivity (HOMA-IR) and *Akkermansiaceae*, *Christensenellaceae*, and *Tanerellaceae*. It also suggests the possibility of a beneficial modulation of the latter two intestinal taxa by a diet high in vegetables and fiber, and low in processed meat.

**Conclusions:**

Overall, our results suggest that the gut microbiome remains stable and highly individual-specific under dietary calorie restriction.

**Trial registration:**

The trial, including the present microbiome component, was prospectively registered at ClinicalTrials.govNCT02449148 on May 20, 2015.

**Supplementary Information:**

The online version contains supplementary material available at 10.1186/s13073-022-01030-0.

## Background

Obesity is an important risk factor for chronic diseases such as type 2 diabetes (T2D), cardiovascular diseases (CVD), and several types of cancer [1]. Aside from well-established mechanisms such as obesity-induced inflammation, alterations in sugar and lipid metabolism, and steroid hormone signaling [2–4], imbalances in the composition of the gut microbiome have also been linked to the progression of obesity and its cardio-metabolic sequelae [5]. Obesity has been associated with a lower abundance of gut bacteria in the *Bacteroidetes* phylum, in contrast to a higher abundance of *Firmicutes* [5]. Furthermore, gut bacteria-dependent turnover of metabolites, e.g., short-chain fatty acids (SCFAs) [6], secondary bile acids [7], trimethylamine-N-Oxide (TMAO) [8] or amino acids [9], acting locally or systemically, may mediate the links between the gut microbiome, obesity, and obesity-associated chronic diseases.

Dietary weight loss may lead to improvements in host metabolism and alleviate the risk of possible obesity-associated comorbidities. Despite evidence to suggest that these benefits are due to modulations of the gut microbiome and related metabolites upon calorie restriction [10–12], findings on the effects of dietary weight loss on the composition and function of the microbiome from randomized trials have been equivocal [13]. This may be attributable to small sample sizes, short trial durations, and diverse microbiome assessment techniques in previous studies, but also to the drastic and restrictive dietary methods used to achieve weight loss in many intervention studies [13, 14]. Moreover, distinguishing transient variability in gut microbiome composition [15, 16] from intervention-associated changes has been challenging since the majority of earlier studies lacked repeated gut microbiome assessments. In addition, it is not known whether some reported initial changes in gut microbiome composition after weight loss persist in the long term, and to what extent this relates to variations in body composition, anthropometric, and clinical biomarkers as well as circulating metabolites.

While mixed results have been reported from the abovementioned studies on different types of continuous calorie restriction (CCR) and the microbiome, intermittent calorie restriction (ICR) has recently been shown to improve several health outcomes in animal models, possibly via effects on the microbiome [17–21]. However, despite these promising findings from animal models and first smaller studies among humans [13], there is a lack of well-powered trials on ICR in relation to the microbiome. Thus, in the present study, we investigated whether ICR (operationalized as the 5:2 diet) or CCR induced alterations in the gut microbiome and to which extent these were associated with overall weight loss irrespective of the dietary intervention in overweight or obese adults. To this end, we analyzed repeated fecal samples over 1 year, i.e., at baseline, 12, 24, and 50 weeks with 16S rRNA gene sequencing and also performed repeated targeted profiling of gut microbiome-related metabolites in the circulation. This study was conducted using data and samples of the HELENA Trial (NCT02449148), a randomized controlled trial undertaken to compare the metabolic effects of ICR vs. CCR [22].

## Methods

### Study design and participants

**Fig. 1:**
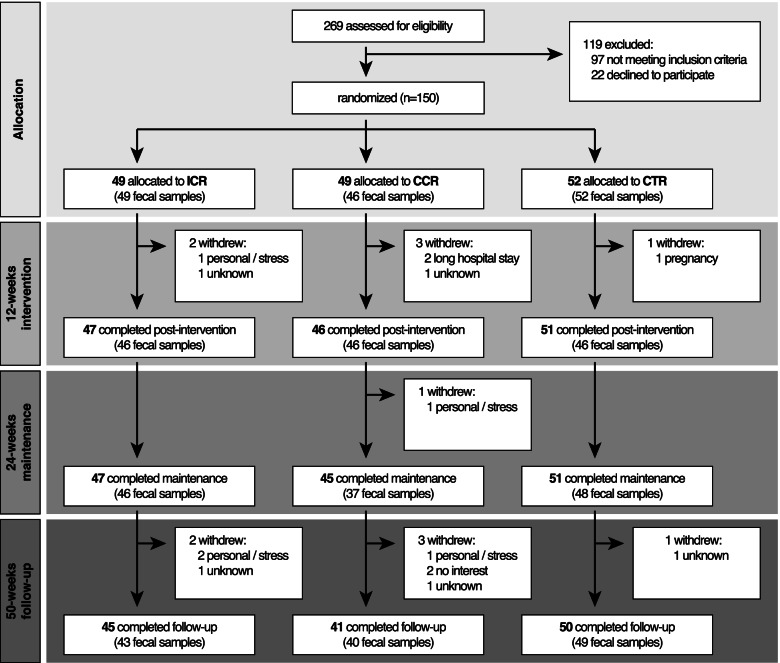
Modified CONSORT diagram showing the flow of participants in the HELENA Trial. ICR – intermittent calorie restriction; CCR – continuous calorie restriction; CTR – control group; CONSORT – consolidated standards of reporting trials

The protocol of the HELENA Trial, a parallel-arm randomized controlled trial which included a 12-week controlled intervention phase with additional dietary counselling, followed by a 12-week maintenance phase (week 24) and a final observational follow-up phase of 26 weeks (week 50) (Fig. 1) has previously been published [23]. Briefly, the trial recruited 150 overweight or obese (BMI > 25 and < 40 kg/m^2^), non-smoking adults (age range 35–65 years, 50% women), free of any gastrointestinal tract diseases as well as major chronic diseases such as CVD or diabetes in Heidelberg, Germany, in 2015. Recruitment was carried out from 6th May, 2015, to 17th May, 2016. At the time of enrolment, none of the participants were taking antibiotics. Participants were randomly assigned to one of three groups, i.e., an intermittent calorie restriction (ICR) (*n* = 49), a continuous calorie restriction (CCR) (*n* = 49), or a control group (CTR) (*n* = 52) over a 50-week period in a 1:1:1 ratio. Overall, fecal samples were obtained from 147 participants at baseline (Fig. 1). In the ICR group, participants reduced calorie intake by ~75% on two non-consecutive days of the week (so-called “5:2 diet”) whereas those in the CCR group reduced daily calorie intake by 20% [22]. All participants (ICR, CCR, and CTR) were advised to adhere to the dietary recommendations of the German Nutrition Society (DGE e.V.), i.e., 55% energy from carbohydrates, 15% from protein, and 30% from fat [24]. The results on the effects of ICR vs. CCR vs. CTR with respect to the pre-specified primary endpoints, i.e., changes in the expression of 82 pre-selected genes in subcutaneous adipose tissue (SAT), as well as secondary endpoints, i.e., anthropometric, body composition, and routine metabolic blood biomarkers, have already been published [22]. There were no differential effects of ICR and CCR [22]. The present analyses on changes in clinical parameters (secondary endpoints) as well as microbiome and metabolites (exploratory endpoints) across the initial trial arms and by overall weight loss were pre-specified in the study protocol of the HELENA Trial [23]. However, all exploratory analyses of the gut microbiome beyond the comparisons across trial arms and weight-loss quartiles had a post hoc character.﻿ These analyses include (i) association tests of gut microbial families with body composition, clinical markers, and dietary intake, (ii) the prediction of subsequent weight loss from baseline microbiome features, as well as (iii) comparisons of microbiome data from the Helena Trial with that from published studies. The ethics committee of the Heidelberg University Hospital (Heidelberg, Germany, Reference: vote S299-2014) as well as the EMBL Bioethics Advisory Committee (Reference: 2020-022) approved the trial, which was designed and conducted in agreement with the principles of the World Medical Association’s Declaration of Helsinki [25]. All participants signed informed consent forms before enrolment. The trial, including the present microbiome component, was registered at clinicaltrials.gov (NCT02449148).

### Dietary assessment

Dietary intake was assessed using food records for 7 consecutive days. Information on the time and location of food intake, portion sizes, and details on the characteristics of the meals/foods consumed were obtained with the 7-day food records [22]. Pictured portion size books were provided to all study participants in order to aid with portion size estimation. Energy and nutrient intake were analyzed based on the German Nutrient Database (Bundeslebensmittelschlüssel, version 3.02) using the software PRODI 6.8 (Nutri-Science GmbH, Hausach, Germany). Daily energy and nutrient intake were estimated as the averages of all reported days [22].

### Blood sample collection and biochemical analyses

Measurements of routine metabolic biomarkers were carried out at the central laboratory of the Heidelberg University Hospital by clinical standard assays right after fasting blood sampling at baseline, and after 12, 24, and 50 weeks after the start of the trial. Fasting blood samples were subsequently aliquoted and immediately stored at − 80 °C at the Biobank of the National Center for Tumor Diseases (NCT), Heidelberg, Germany, until further biochemical analyses. The concentrations of routine blood-based biomarkers such as LDL cholesterol, HDL cholesterol, total cholesterol, triglycerides, glucose, GGT, ALT, AST, and of serum biomarkers (adiponectin, CRP, IL-8, IL-6, IFN-γ, TNFα, resistin, leptin, insulin, IGF-1) were measured as described in detail previously [22]. The collection of all biospecimens, and subsequent biochemical analyses in the HELENA Trial was conducted in line with standard operating procedures [22].

Plasma SCFA concentrations were determined by high-performance liquid chromatography-tandem mass spectrometry (HPLC-MS/MS) at the Institute of Agricultural and Nutritional Sciences, Martin Luther University Halle-Wittenberg using a modified form of the methods by Chan et al. [26] and Zeng and Cao [27]. Further details of the measurements of the SCFAs have been described previously in a related publication [28].

Targeted metabolomics analysis of plasma acylcarnitines and amino acids as their butyl esters was performed on dry blood spot samples by tandem mass spectrometry (MS/MS), using a triple quadrupole tandem mass spectrometer with [^2^H_3_] methionine and [^2^H_5_] phenylalanine as internal deuterated standards as previously described [29]. Plasma acylcarnitine and amino acid analysis were carried out at the *Stoffwechsellabor* (new-born screening laboratory), University Hospital Heidelberg, Germany.

The concentrations of bile acids were measured at the Analytical Unit of the Health Research Institute Hospital La Fe (Valencia, Spain) by means of a validated ultra-performance liquid chromatography/multiple reaction monitoring/mass spectrometry (UPLC-MRM-MS) method, details of which have already been published [30]. Liquid chromatography-high-resolution mass spectrometry was used to measure TMAO, betaine, and choline levels in plasma at the Institute of Clinical Chemistry, University Hospital Zurich (Zurich, Switzerland) as described previously [31].

### Fecal sample collection and DNA extraction

Fecal samples were collected from participants at four different time points, i.e., baseline and weeks 12, 24, and 50 into sterile RNA*later™*-filled tubes (Thermo Fisher Scientific, Germany) and stored at − 80°C at the Biobank of the National Center for Tumor Diseases, Heidelberg, Germany, until DNA extraction. Bacterial genomic DNA was extracted from fecal samples according to the manufacturer’s protocol using the AllPrep® PowerFaecal® DNA/RNA Kit (QIAGEN, Hilden, Germany). DNA from all fecal samples belonging to the same participant were extracted in the same run. In brief, approximately 200 mg of feces were added to lysis tubes containing glass beads and a lysis buffer. The tubes were then put into a Precellys® 24 Homogenizer (Bertin instruments, France) for mechanical bacterial cell lysis, which was performed twice—45 s each with an intermediate incubation period of 5 min. Genomic DNA was bound to silica membranes in spin columns, washed, and finally eluted into 1.5-ml tubes after a final incubation period of 1 min. The Qubit® 2.0 Fluorometer with broad range assay (Thermo Fisher Scientific, Germany) was used to quantify DNA concentrations (ng/μl) according to the manufacturer’s instructions. Genomic DNA was immediately stored at − 20 °C until further analyses.

### Library preparation and 16S rRNA gene sequencing

In the first stage of a two-stage PCR protocol, the V4 region of the 16S rRNA bacterial gene was targeted for selective PCR amplification using V4 region-specific 16S rRNA primers 515F (GTGCCAGCMGCCGCGGTAA) and 806R (GGACTACHVGGGTWTCTAAT) [32]. For each DNA sample, a volume of 3 μl (~ 10 ng) was used as a template in a total PCR volume of 12 μl in the first-stage PCR with conditions of an initial denaturation step at 98 °C for 2 min, followed by 20 cycles of a three-step process comprising (i) denaturation at 98 °C for 10 s (ii) annealing at 65 °C for 20 s (iii) extension 72 °C for 20 s. The first-stage PCR ended with a final extension step at 72 °C for 2 min.

In the second PCR amplification, a volume of 3 μl PCR product from the first PCR step was used as a template in a ~ 12 μl total PCR volume. The PCR conditions and steps were similar to the first, but with 15 rather than 20 cycles. In order to allow for pooling/multiplexing of samples for the 16S rRNA gene sequencing after the second PCR step, each sample was amplified with a unique 12-base barcode primer (Nextera® barcodes, Illumina Inc., USA). Repeat DNA samples from the same participant were amplified in the same PCR run.

**Fig. 2:**
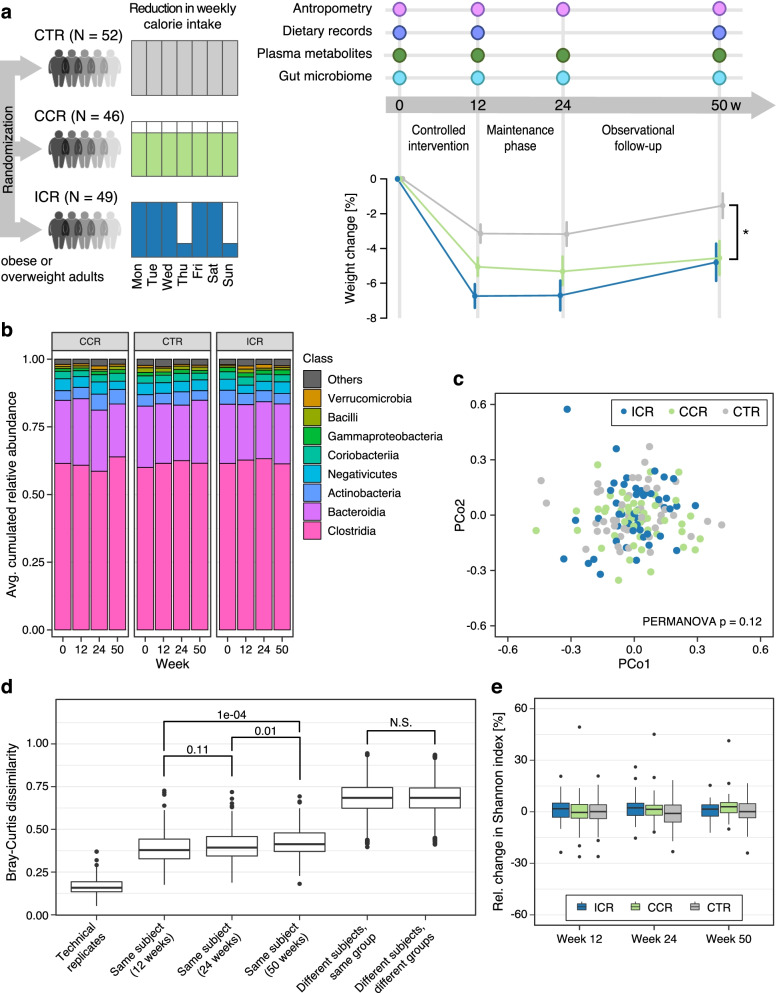
Overview of the study and intervention effects on the gut microbiome composition. **a** Graphical overview of the HELENA Trial design showing details of all data collected over the course of the trial. In total, 150 overweight/obese adults were randomly assigned to the ICR (5 days eucaloric diet, 2 days of ~25% energy requirement), CCR (daily reduction of ~20% of caloric intake) or CTR group, 147 of them provided stool samples at baseline. Anthropometric measurements were performed for all participants. Blood samples were collected at all study timepoints for the assessment of plasma/serum concentrations of routine biomarkers. Dietary information was collected at baseline, week 12, and week 50. The amount of weight loss was significantly higher among ICR and CCR participants compared to CTR participants at all study timepoints, as previously published by Schübel et al. [22]. Differences in weight change across intervention groups at each timepoint was assessed using linear mixed models adjusted for age and sex, with participant identifiers as random effects in the model. A significant difference, i.e., *p* < 0.05 is indicated by (*). ICR – intermittent calorie restriction; CCR – continuous calorie restriction; CTR – controls. **b** The gut microbiota was predominantly composed of bacteria belonging to *Clostridia* followed by *Bacteroidia* as shown by stacked bar charts of taxonomic classes across timepoints and subdivided by intervention group. **c** Non-metric multidimensional scaling (NMDS) plots of Bray-Curtis dissimilarity distances based on ASV relative abundances between samples after 12 weeks of the intervention. Each point represents the microbial community of a sample, and the colour indicates the intervention group to which it belongs. NMDS plots do not show clustering by intervention groups and PERMANOVA does not indicate a statistically significant difference between groups. **d** Intra-individual variability (same subject compared across time points) was significantly lower compared with inter-individual variability (different subjects, same group and different subjects, different groups). Technical replicates in this study showed a very low variation in Bray-Curtis dissimilarity distances based on ASV relative abundances, attesting to a high technical reproducibility. Statistical significance as indicated above boxplots was assessed by Wilcoxon signed-rank test. **e** There was no significant intervention effect on overall gut microbiota alpha diversity (Shannon Index) across all intervention timepoints. All boxplots show the interquartile ranges (IQRs) as boxes, with the median as a black horizontal line and the whiskers extending up to the most extreme points within 1.5-fold IQR. Additional file 3: Source data 2

The success of amplification of each sample was assessed by visualization of bands on a 2.5% agarose gel against a 100-bp ladder. Samples that did not amplify were repeated, after which all samples were pooled. A clean-up of the pool was performed with SPRIselect® magnetic beads (Beckman Coulter Inc., USA), using 0.8 left side size selection according to the manufacturer’s instructions. The cleaned pool was again quantified using the Qubit® 2.0 Fluorometer with high sensitivity assay. The Agilent 2100 Bioanalyzer® system (Agilent Technologies, USA) was used to assess the average size distribution, the quality, and again the concentration of the pooled sample. Afterwards, a 2 × 250 paired-end sequencing of the V4 region of the 16S rRNA gene was carried out on the Illumina Miseq platform (Illumina, Inc., USA) according to the manufacturer’s instructions as previously outlined [33]. Sequencing was performed in two batches, but all samples from the same participant were sequenced in the same batch. The library preparation and 16S rRNA gene sequencing for a selection of the samples were performed twice for the technical validation of both steps (Fig. 2d). Library preparation and 16S rRNA gene sequencing were carried out at the Genomics Core Facility of the European Molecular Biology Laboratory (EMBL), Heidelberg.

### Analysis of 16S rRNA amplicon sequences

After sequencing, the raw sequence reads were demultiplexed based on their unique primer barcode identifiers using MiSeq instrument software and prepared for further downstream processing and analysis. The demultiplexed sequence reads were processed with DADA2 (Divisive Amplicon Denoising Algorithm), a tool able to identify the original sequences that generated the observed 16S sequences, known as amplicon sequence variants (ASVs) [34]. Using DADA2, raw sequence reads were quality filtered and denoised, after which paired-end reads were merged, and chimeric sequences discarded. A total of 13,767,541 reads were generated after Miseq paired-end sequencing, and 11,546,617 reads remained after filtering, denoising and chimeric sequences removal. The reads were grouped into amplicon sequence variants (ASVs) based on sequence similarity, and subsequently rarefied/subsampled to an equal depth of 5000 sequences per sample. This was performed to account for variability in sequencing depth. Only three samples were excluded after rarefaction. Standard quality control checks identified a probable case of sample swap involving 2 samples, which was resolved by relabeling based on Euclidean distance clustering of the samples (Additional File 1: Figure S1).

Alpha diversity (within-sample species diversity) at baseline and all other time points was estimated based on the Shannon index. For the assessment of gut microbiome compositional differences between the intervention groups, i.e., beta diversity, non-metric multidimensional scaling (NMDS) plots based on the Bray-Curtis dissimilarity matrices were generated on ASV relative abundances of samples for visual inspection of clustering and multivariate testing.

Taxonomic annotation at phylum, class, order, family, and genus levels was performed by matching pre-processed sequence reads against the SILVA rRNA gene database [35]. For each taxonomic rank at each specific time point(s), only bacteria clades that were present in at least 5% of the samples were retained for further analyses. The read counts were further converted to relative abundances. An unbiased assessment of the technical reproducibility using technical replicates of samples showed a low variability in the Bray-Curtis dissimilarity distances between technical replicates in this study (Fig. 2d; *Technical replicates*), which points to high reproducibility.

*Prevotella-to-Bacteroides* ratio (P/B ratio) was calculated as the summed relative abundance of the ASVs that map to *Prevotella* (“*Prevotella*”, “*Prevotella_2*,” etc.), divided by the cumulative relative abundance of the ASVs that map to the *Bacteroides* genus in Silva. In cases where the sum of *Prevotella* was zero, the P/B ratio was set to 1/10,000. The P/B ratio was set to 10,000 when the sum of *Bacteroides* relative abundances was zero. Lastly, when the sum of both *Prevotella* and *Bacteroides* relative abundances was zero, the P/B ratio was set to 1. We subsequently applied a log10 transformation to improve visualization and compared the P/B ratio across intervention groups and also assessed the association of P/B ratio with weight loss.

### Statistical analysis

Data for continuous variables, including changes, are presented as means ± SEM (standard error of the mean), and as frequencies and/or percentages for categorical variables. Relative changes in body composition and in the concentrations of clinical biomarkers and circulating metabolites from baseline to each post-intervention phase, i.e., weeks 12, 24, and 50 were calculated across the initial intervention groups (ICR, CCR, and CTR). Differential changes in metabolite concentrations between the intervention groups at each time point were assessed using linear mixed effect (LME) models. The LME model included age, sex, time, intervention group, and a time-by-intervention interaction term, with participants’ identifier or subject ID set as the random effect. Differences between the intervention groups with respect to changes in the outcomes of interest were considered significant at *p* < 0.05 for the time-by-intervention interaction effect. When the overall time-by-intervention effect was significant, pairwise comparisons were performed, again using LME models as described.

For 16S rRNA data, bacteria relative abundances were log-transformed, after zeros had been replaced with a small number (pseudocount of 1E−04) to avoid taking the log of zero. Log-fold changes in bacteria relative abundances at each post-intervention time point were computed relative to baseline. Non-parametric Kruskal-Wallis tests were performed to assess the differences in the relative abundance of each individual gut microbial taxon between the intervention groups at baseline.

The differential effects of interventions on individual gut microbiome relative abundance and diversity at weeks 12, 24, and 50 from baseline were evaluated using LME models as already described for the intervention effects on routine clinical markers and circulating metabolites. When the difference in microbiome relative abundance across all intervention groups was significant, pairwise comparisons were performed, again with LME models with FDR corrections using the Benjamin-Hochberg FDR correction method [36]. The associations of changes in the abundances of individual bacteria which were found to be differentially abundant at specific timepoints with changes in clinical and dietary variables were also assessed using Spearman’s correlations. An association was considered to be statistically significant at an FDR-corrected *p* < 0.05.

In order to examine the unique contribution of baseline bacterial abundance on post-intervention abundances, i.e., the proportion of variance in post-intervention abundance explained by baseline abundances, we fitted linear regression models with age, sex, intervention group, and baseline bacterial relative abundance as independent variables, and each post-intervention abundance as dependent variable. In each model, we examined the coefficient of partial determination or partial *R*-squared of each independent variable and their significance in the model.

To further analyze differences in the overall gut microbiome composition between the intervention groups post-intervention, permutational analysis of variance (PERMANOVA) (permutations = 999) was performed using the adonis function in the vegan R Package [37] on Bray-Curtis dissimilarity matrices generated between samples at each timepoint.

In a further analyses step, we examined the effect of overall weight loss irrespective of the dietary regimen, i.e., ICR, CCR, or CTR on gut microbiome composition. The changes in gut microbiome abundances, alpha diversity, and overall composition across the time points were then profiled with respect to overall weight loss using LME models. The changes in metabolic parameters and circulating metabolites were also evaluated according to overall weight loss using LME models. Trends for linear associations between weight loss and changes in dependent variables, i.e., bacterial relative abundances or concentrations of circulating metabolites, were modelled with weight loss as a continuous parameter.

In order to examine associations between core families (prevalence > 50%) within the gut microbiome and body composition, clinical markers, and also dietary intake, we used LME models with the R package *lmerTest* [38]. LME models include both fixed and random effects and can therefore uncover associations in data which are not independent. In our case, we used LME modelling to find common associations between bacterial families and clinical markers (as fixed effects), including the participant ID as random effect to account for the non-independence of repeated measurements. The formula for the LME model was *family ~ marker + (1 | Participant_ID)*, allowing for a different intercept for each individual. This way, inter-individual differences at baseline are accounted for, as a means of dealing with the well-established strong inter-individual variation of gut microbiome composition [39]. In a second analysis, we also included the weight loss quartile as a modifying effect by adjusting the formula to: *family ~ marker + (1 + weight_loss_quartile | marker) + (1 | Participant_ID)*. For visualization of the results, we extracted the participant-specific intercepts and used them to adjust the log-transformed family abundances. *P* values from LME models were corrected with the Benjamini-Hochberg method [36].

To examine whether gut microbiota profiles at baseline were predictive of weight loss outcomes, we pooled participants across all intervention groups to identify associations between microbiome features and weight loss at later time points. At each time point, participants were grouped into weight loss quartiles based on the percentage of weight lost compared to the initial time point. We then used linear models as well as ROC analysis to find associations between the top and bottom weight lost quartile and different microbial features: we investigated (i) the baseline abundance of bacterial genera, (ii) the baseline *Prevotella*-to-*Bacteroides* ratio, (ii) baseline gut microbiota richness, and (iv) microbiome plasticity (defined as Bray-Curtis dissimilarity between the first and second time point). To compare our findings to those reported in the study by Grembi et al. [40], which investigated associations between initial microbiome composition and weight development over 1 year, we downloaded the raw reads of all technically comparable samples (paired-end sequencing, corresponding to the validation cohort in Grembi et al., *n* = 56, SRA project ID=PRJNA542910) and conducted the same profiling procedure described above (DADA2 plus annotation to the Silva database).

For all analyses, visual inspection of histograms and normal probability plots, as well as Kolmogorov-Smirnov tests, were performed when relevant, for the assessment of normality assumptions. All statistical analyses were two-tailed.

All statistical analyses were carried out using R statistical software [41].

## Results

### Baseline characteristics of participants

Overall, 147 participants (74 men and 73 women), out of the 150 enrolled, provided fecal samples at baseline (ICR, *n* = 49, CCR, *n* = 46, CTR, *n* = 52) (Fig. 1, see also Fig. 2), indicative of high compliance at baseline, which was also observed at subsequent timepoints. Their mean age at baseline was 50.3 ± 7.9 years, with an average BMI of 31.4 ± 3.7 kg/m^2^ across all groups (Table 1, see also Additional file 2: Table S1 for further baseline characteristics).

**Table 1:** Baseline characteristics of study participants according to intervention group^a^

	ICR (*n* = 49)	CCR (*n* = 46)	Control (*n* = 52)	*p*^b^
Sex, *n* (%)				
Men, *n* (%)	25 (51.0%)	24 (52.2%)	25 (48.1%)	
Women, *n* (%)	24 (49.0%)	22 (47.8%)	27 (51.9%)	
Age, years	49.4 ± 9.0	50.8 ± 7.8	50.6 ± 7.1	0.71
Weight, kg	96.4 ± 15.8	93.0 ± 16.0	93.3 ± 13.3	0.39
BMI, kg/m^2^	32.0 ± 3.7	31.2 ± 3.9	31.1 ± 3.6	0.46
Subcutaneous adipose tissue, cm^3^	12822 ± 4267	12167 ± 3985	11945 ± 3845	0.55
Visceral adipose tissue, cm^3^	4818 ± 1889	5012 ± 2183	4943 ± 2267	0.96
Systolic blood pressure, mmHg	139.4 ± 18.7	136.3 ± 16.9	136.0 ± 12.5	0.47
Diastolic blood pressure, mmHg	87.2 ± 9.9	87.4 ± 8.6	87.8 ± 7.3	0.93
Glucose, mg/dL	92.7 ± 7.5	94.3 ± 7.6	93.5 ± 7.4	0.69
HOMA-IR	2.7 ± 1.3	3.0 ± 1.8	3.0 ± 1.8	0.58
Triglycerides, mg/dL	130.0 ± 83.8	122.5 ± 67.1	145.0 ± 85.5	0.31
Cholesterol, mg/dL	205.0 ± 30.8	204.6 ± 39.8	211.8 ± 36.1	0.42

^a^*n* = 147. Values are means ± SD. ICR — Intermittent calorie restriction, CCR — Continuous calorie restriction

^b^ANOVA comparison between intervention group for all variables except sex. *p*-value for sex is from chi-square test for the comparison of the distribution of sexes across intervention groups. Values are reported only for participants gut microbiota data at baseline

As expected, there were no differences in core baseline characteristics by group. When categorizing the sample by quartiles of overall weight lost during the trial, there were no significant differences at baseline either (Table 2, see also Additional file 2: Table S2 for further characteristics).

**Table 2:** Baseline characteristics of participants according to weight loss quartiles^a^

	Q1 (***n*** = 36)	Q2 (***n*** = 35)	Q3 (***n*** = 36)	Q4 (***n*** = 36)	***p***^**b**^
Sex, *n* (%)					
Men	16 (44.4)	21 (60.0)	17 (47.2)	18 (50.0)	
Women	20 (55.6)	14 (40.0)	19 (52.8)	18 (50.0)	
Age, years	51.0 ± 6.3	51.5 ± 8.1	51.2 ± 7.8	47.4 ± 8.3	0.11
Weight, kg	94.2 ± 15.8	94.3 ± 14.1	93.3 ± 15.5	95.0 ± 14.2	0.97
BMI, kg/m^2^	32.1 ± 4.1	31.1 ± 3.7	30.9 ± 3.4	31.5 ± 3.7	0.53
Subcutaneous adipose tissue, cm^3^	13107 ± 4620	11153 ± 2842	12118 ± 3933	12871 ± 4047	0.18
Visceral adipose tissue, cm^3^	5253 ± 2249	5037 ± 2191	4836 ± 2006	4720 ± 2038	0.75
Systolic blood pressure, mmHg	139.6 ± 11.0	132.7 ± 13.8	136.6 ± 14.4	140.0 ± 21.9	0.14
Diastolic blood pressure, mmHg	90.1 ± 8.1	86.3 ± 8.0	87.3 ± 7.7	86.9 ± 9.8	0.19
Glucose, mg/dL	93.4 ± 7.9	93.4 ± 6.8	94.8 ± 6.8	91.8 ± 8.0	0.40
HOMA-IR	3.4 ± 1.9	3.0 ± 1.8	2.5 ± 1.2	2.6 ± 1.3	0.09
Triglycerides, mg/dL	139.4 ± 64.9	137.8 ± 90.0	143.9 ± 93.2	108.3 ± 53.5	0.20
Cholesterol, mg/dL	211.4 ± 34.1	202.3 ± 36.4	214.4 ± 36.0	203.2 ± 34.5	0.36

^a^*n* = 143. Values are means ± SD. ^b^ANOVA comparison between all weight loss quartiles for all variables except sex. *p*-value for sex is from Chi-square test for the comparison of the distribution of sexes across all quartiles at baseline. Q1, Q2, Q3, and Q4—weight loss quartiles 1, 2, 3, and 4 respectively. Values are reported only for participants with gut microbiota data at baseline and also had weight measurements at week 12, i.e., non-dropouts

Consistent with randomization, there was no significant difference in the relative abundance of bacteria between the intervention groups (ICR vs CCR vs CTR) at baseline (Kruskal-Wallis test; FDR-corrected *p*-value, i.e., *q* value > 0.05 for individual bacteria at all taxonomic levels, for bacterial classes see Fig. 2b). Expectedly, overall gut microbiome composition was dominated mainly by two bacterial classes: *Clostridia* followed by *Bacteroidia* (Fig. 2b), which belong to the two most abundant phyla of gut microbiota, i.e., *Firmicutes* and *Bacteroidetes*, respectively [42].

### Dietary intervention did not cause major changes in gut microbiome composition and diversity

Both ICR and CCR interventions, compared to CTR, resulted in significantly greater reductions in weight over the course of the intervention (Fig. 2a). In contrast, gut microbiome composition remained relatively stable and ICR and CCR did not induce major changes (Fig. 2b). Beta diversity analysis (quantifying between-sample differences) using Bray-Curtis dissimilarity on amplicon sequence variants (ASVs) did not reveal any clustering by intervention groups after 12 weeks (Fig. 2c), as is apparent from visualizations based on non-metric multidimensional scaling (NMDS) (Fig. 2c, PERMANOVA *p* = 0.12). Consistent with this finding, beta diversity at later time points (week 24 and week 50) was not significantly affected by the intervention either (Additional file 1: Figure S2). Likewise, we did not observe an intervention effect on the *Prevotella-to-Bacteroides* ratio (P/B ratio) (Additional file 1: Figure S3).

Instead, we found microbiome composition to primarily vary between individuals irrespective of intervention group. Consistent with previous observational microbiome studies [40, 43], inter-individual differences were substantially higher than intra-individual differences between samples taken from the same subject (Fig. 2d). We moreover observed a slow, but significant, decrease in intra-individual similarity in gut microbiome composition over time (Fig. 2d). However, inter-individual differences between versus within study groups were insignificant (Fig. 2d). Similar to our findings on beta diversity, the relative changes in gut microbiome alpha diversity, assessed by the Shannon index, were not significantly different between the intervention groups at any of the time points either (Fig. 2e, see also Additional file 2: Table S3).

### Intervention affected intestinal *Lactobacillales* abundance

**Fig. 3:**
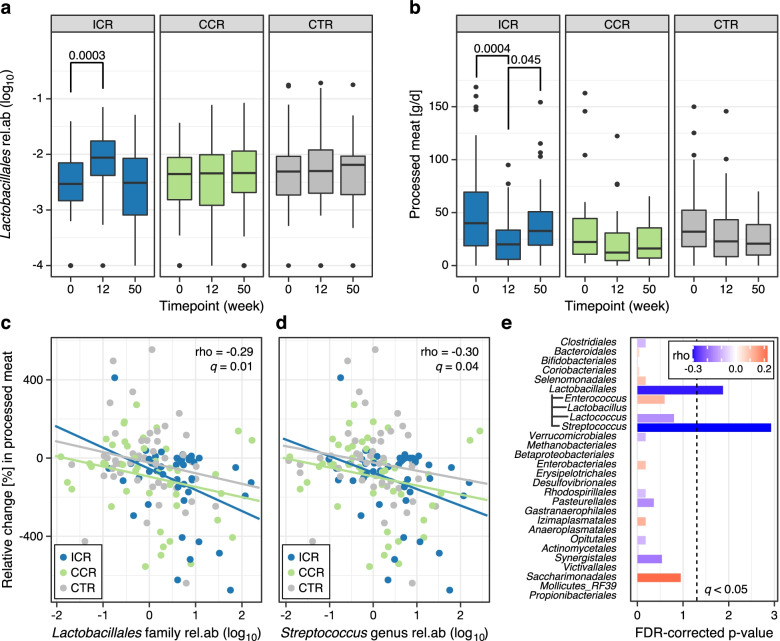
Changes in *Lactobacillales* abundance and processed meat intake, and associations between changes in *Lactobacillales* abundance and processed meat intake. **a** The relative abundance of the order *Lactobacillales* increased in the ICR group after the 12-week intervention but returned to levels similar to baseline at the end of follow-up as shown by boxplots (defined as in Fig. 3); statistical significance was assessed by Wilcoxon signed-rank test. **b** Participants in the ICR group significantly reduced their intake of processed meat during the 12-week intervention period, although intakes increased again at follow-up. In contrast, there was no significant change in the consumption of processed meat in either the CCR or CTR groups as shown by boxplots. **c** The changes in the abundance of *Lactobacillales* after 12 weeks were significantly associated with the changes in processed meat intake (Spearman correlation, *q* = 0.02). Scatter plot showing Spearman correlation between changes in the relative abundance of *Lactobacillales* and changes in processed meat intake. **d** The genus *Streptococcus,* which belongs to the order *Lactobacillales*, also significantly co-varied with the changes in processed meat intake after the 12-week intervention (*q* = 0.02). **e** Abundance changes in *Lactobacillales* and its member genus *Streptococcus* were significantly associated with changes in processed meat intake after 12 weeks. Bar length indicates *q* value (FDR-corrected *p*-value) and effect size is color-coded. Additional file 4: Source data

When assessing changes in the relative abundances of individual bacterial taxa over time, we only found the order *Lactobacillales* to be significantly increased in the ICR group, in contrast to marginal decreases in both the CCR and CTR (Additional file 2: Table S4) right after the intervention period (Fig. 3a). This increase in the ICR group was still detectable for the class of *Bacilli*, to which it belongs (Additional file 2: Table S5). At lower taxonomic ranks within *Lactobacillales*, the family *Streptococcaceae* showed the strongest change after 12 weeks (*q* = 0.12), with median relative changes (25th–75th percentile) of 0.62 (− 0.12,0.89) vs. 0.00 (− 0.56,0.46) vs. 0.00 (-0.51,0.42) for ICR vs. CCR vs. CTR respectively (see also Additional file 2: Table S14).

Given that weight loss did not significantly differ between ICR and CCR (Fig. 2a), we further analyzed whether the differential increases in *Lactobacillales* and *Bacilli* with ICR were attributable to changes in dietary composition. The only dietary item that showed a differential relative change with ICR was processed meat consumption (its decrease during intervention was likely due to higher baseline levels despite randomization, Fig. 3b). In fact, the changes in *Lactobacillales* relative abundance was negatively associated with changes in processed meat intake after 12 weeks (rho = − 0.19, *q* = 0.02, Fig. 3c. See also Fig. 3e). Within the *Lactobacillales* order, a slightly stronger negative association was apparent for the genus *Streptococcus* after 12 weeks (rho = − 0.23, *q* = 0.02, Fig. 3d). Details of changes in dietary intake according to intervention groups is presented in Additional file 1: Figure S4.

With respect to circulating metabolites, we did not observe significant effects of the interventions on the concentrations of plasma bile acids, most amino acids, and acylcarnitines as well as TMAO and its precursors across timepoints, consistent with the lack of significant effects of the interventions on gut microbiome composition in our study.

### Weight loss was associated with changes in metabolic biomarkers but not gut microbiome composition or microbial metabolites

**Fig. 4:**
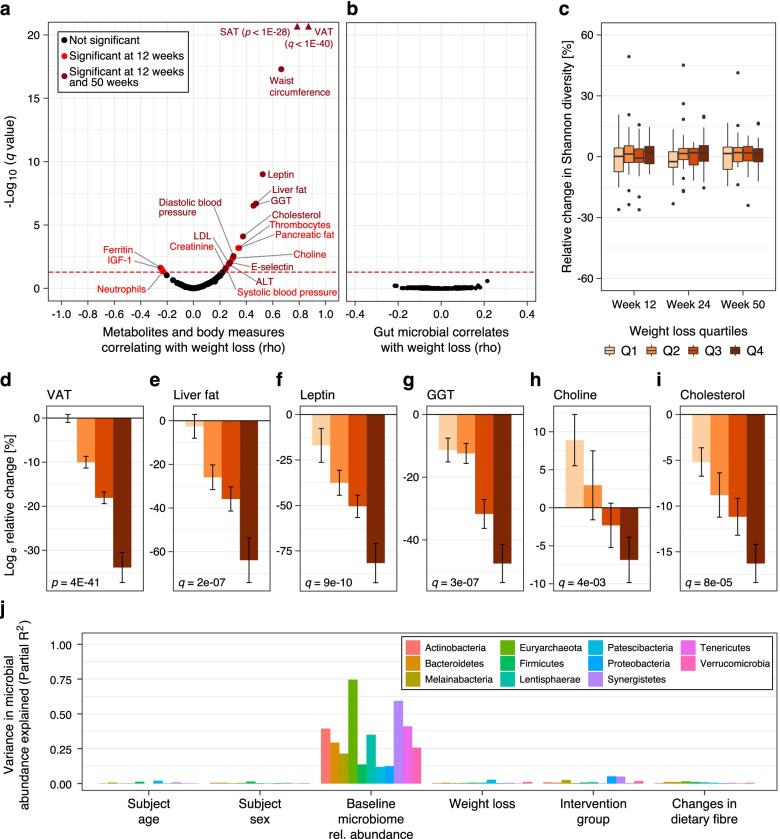
Association of weight loss with anthropometric, clinical markers, metabolic biomarkers and the gut microbiome. **a** Weight loss was significantly associated with decreases in body fat compartments (e.g., VAT and SAT), and routine biomarkers (e.g., cholesterol and LDL), but not with changes in gut microbial and small-molecule metabolites, with the exception of choline. Only variables significantly associated with weight loss are labelled (red dots). Measured metabolites (unlabelled due to a lack of association with weight loss) aside routine biomarkers such as those of glucose metabolism, e.g., insulin, comprised bile acids, SCFAs, TMAO, and its precursors, amino acids, and acylcarnitines, but also inflammatory cytokines (Additional file 5: Source data 4). Variables shown by dark red spots were significantly associated with weight loss also at follow-up, i.e., week 50. **b** The changes in weight after 12 weeks were not significantly associated with any individual bacterial taxon across all taxonomic levels, after FDR correction (all taxonomic levels included in the plot) **c** There was no differential association of weight loss with gut microbial alpha diversity across all timepoints. Boxplots show changes in alpha diversity over time according to quartiles of weight loss (Q1—quartile 1, Q2—quartile 2, Q3—quartile 3, and Q4—quartile 4, see Fig. 2 for definition of boxplots). Noticeably, metabolic improvements based on the changes in anthropometric and body composition parameters as well as blood concentrations of some biomarkers were greater among participants in Q4 who achieved the highest amount of weight loss after 12 weeks (See Additional file 2: Table S8). As also indicated by the volcano plot in **a**, there was a significant linear association between weight loss and changes in **d** visceral adipose tissue, **e** liver fat, **f** leptin, **g** gamma-glutamyl transferase (GGT), **h** choline, and **i** cholesterol after the 12-week intervention period. **j** Baseline abundances, rather than intervention group or weight loss accounted for the largest variation in post-intervention relative abundance of bacteria (here shown at phylum level). Bar plot showing the variations in phyla relative abundance (see colour key) after 12 weeks of intervention accounted for by age at recruitment, sex, baseline phyla abundance, and weight loss (%). Models were generated separately for weight loss and intervention, while partial R-squares of age, sex, and baseline abundances are from the model including weight loss. Additional file 5: Source data 4

To assess whether the potential metabolic improvements that may accompany dietary weight loss were linked to the gut microbiome, we investigated the effect of overall weight loss, irrespective of the dietary regimen, on plasma concentrations of routine biomarkers as well as gut microbiome composition and concentrations of microbial-related metabolites in circulation. Weight loss was strongly associated with variations in body composition parameters such as visceral adipose tissue (VAT), subcutaneous adipose tissue (SAT), and liver and pancreatic fat after 12 and 50 weeks (Fig. 4a). Moreover, we found highly significant metabolic improvements associated with weight loss: established biomarkers of lipid metabolism (cholesterol, low-density lipoprotein (LDL) and leptin), liver, and kidney function (ALT and GGT) as well as cardiovascular health (thrombocytes and E-selectin as well as blood pressure) were all positively linearly associated with weight loss (Fig. 4a, d–i and Additional file 2: Table S6).

However, despite the clear metabolic improvements mentioned above, there were no significant associations between overall weight loss and changes in bacterial relative abundances at any taxonomic level after the 12-week intervention (Fig. 4b). Likewise, bacterial alpha diversity was not significantly associated with weight loss at any time post-intervention (Fig. 4c, see also Table S7), nor did we observe any association with the P/B ratio (pre- or post-treatment) or changes therein (Additional file 1: Figure S3), in contrast to what was reported previously [44, 45]. The notion of metabolic improvements occurring independently of gut microbial changes was further supported by the lack of weight loss effects on circulating microbial metabolites, such as plasma SCFAs, bile acids, TMAO, and betaine after 12 and 50 weeks (Fig. 4a, see also Tables S8, S9, S10). However, choline concentrations were observed to decrease over time (Fig. 4h).

When jointly analyzing factors influencing gut microbiome composition, we found baseline microbial abundance to account for the largest proportion of variance in post-intervention abundances by far, with only minimal contributions from intervention group, weight loss, and other sources investigated (as illustrated for bacteria at the phylum level in Fig. 4j). The absence of observable shifts over time due to both intervention and weight loss was further confirmed by ordination analysis revealing predominant clustering by individuals (Additional file 1: Figure S5).

### Baseline gut microbiota features predictive of weight loss

**Fig. 5:**
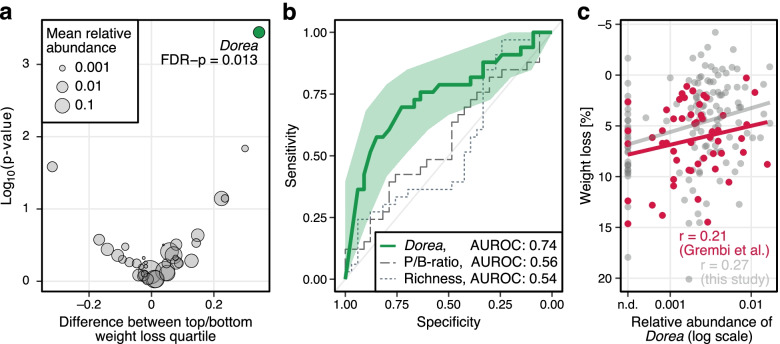
Association of baseline gut microbiota composition with weight loss. A higher abundance of the genus *Dorea* at baseline may be associated with a difficulty in losing excess weight. **a** Volcano plot showing the difference in the relative abundance of *Dorea* between participants in the highest quartile of weight loss and those in the lowest at baseline. Baseline *Dorea* abundances were significantly higher among participants in the lowest quartile of weight loss. **b** Baseline *Dorea* abundances rather than *Prevotella*-to-*Bacteroides* ratio or gut microbiota richness was predictive of post-intervention weight loss. AUROC showing how the aforementioned parameters were predictive of weight loss. **c** The relative abundance of *Dorea* at baseline was positively associated with weight loss, i.e., a higher abundance corresponds to a lower amount of weight loss. A scatter plot showing the association between the relative abundance of *Dorea* at baseline and weight loss after 12 weeks. Additional file 6: Source data 5

When exploring in more detail if baseline microbial features would be predictive of post-intervention weight loss, we observed the abundance of *Dorea* at baseline, as the only one among all genera tested, to significantly differ among participants in the lowest compared to the highest quartile of weight loss after 12 weeks (FDR-corrected *p* = 0.013, Fig. 5a). Low *Dorea* abundance at baseline was moderately predictive of weight loss after 12 weeks when evaluated as a predictive biomarker for classifying participants into the lowest versus the highest weight loss quartiles with an area under the receiver operating characteristic curve (AUROC) of 0.74 (Fig. 5b). Predictive accuracy remained similar when evaluated against weight loss at later time points (Figure S6). We further compared *Dorea* abundance to other biomarkers proposed earlier as predictors of weight loss, such as the *Prevotella*-to-*Bacteroides* (P/B) ratio, or gut microbiota richness [46]. However, neither the P/B ratio (AUROC: 0.56) nor microbiota richness (AUROC: 0.54) were predictive of weight loss in the HELENA Trial (Fig. 5b). We further investigated whether lower *Dorea* abundance at baseline would also be associated with higher weight loss in an independent dataset (Fig. 5c) collected by Grembi et al. [40] in a dietary intervention study with participant characteristics similar to ours. Also in this external data set, we observed a similar association (Pearson’s *r* = 0.21 compared to 0.27 in the Helena Trial), which however did not reach statistical significance (*P* = 0.13) (Fig. 5c). We finally also investigated whether gut microbiota plasticity, as proposed by Grembi et al. and defined as the variability in the gut microbiota composition estimated with a β-diversity metric [40], was associated with weight loss. As multiple pre-intervention microbiome samples were not available in the Helena Trial, we assessed Bray-Curtis dissimilarity-based plasticity between baseline and week 12, i.e., T0–T1 as a predictor of long-term weight loss (i.e., at week 50). In contrast to *Dorea* baseline abundance, microbiota plasticity was not predictive of long-term weight loss in the Helena Trial (AUROC: 0.56, Figure S6).

### Gut microbial families associated with clinical biomarkers, body composition, and dietary intake

**Fig. 6:**
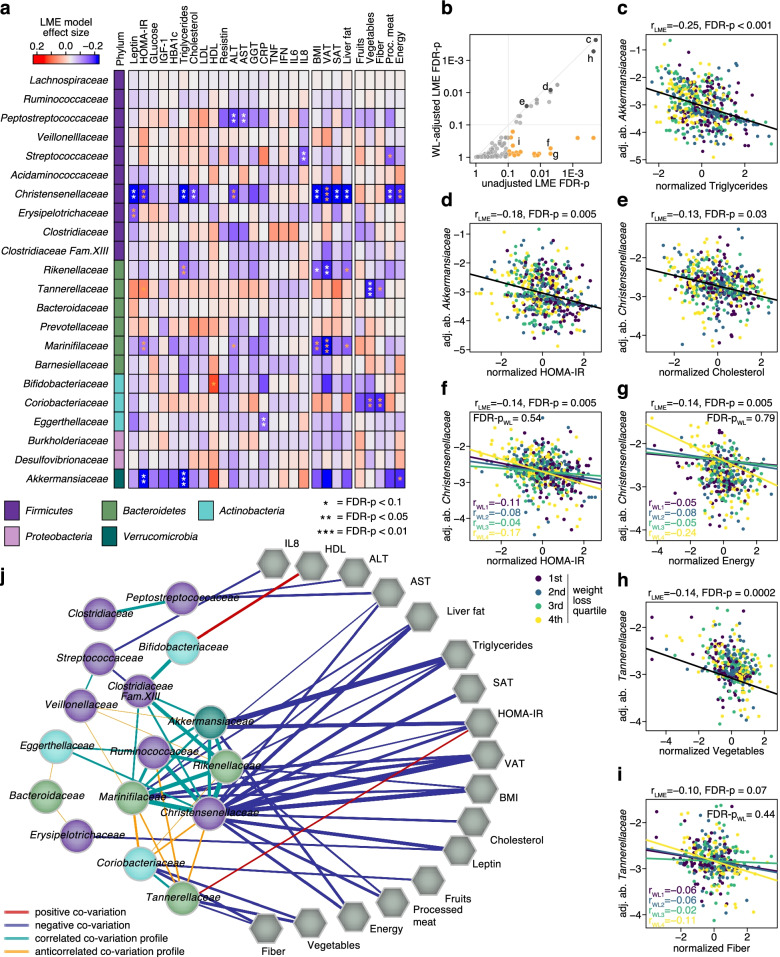
Association of the microbiome with anthropometric measurements, metabolic biomarkers, and dietary intake across timepoints. Several core families within the gut microbiome significantly co-vary with some of the clinical biomarkers, body composition measures, and dietary intake assessed in the trial. **a** Heatmap showing the strength of association calculated irrespective of weight loss quartile or outcome. Phylum affiliation for each family is indicated as a colour strip on the left. Significance for each association after FDR correction is indicated by asterisks (*q* < 0.1 (*), *q* < 0.05 (**), and *q* < 0.01 (***)). Asterisks colored yellow were no longer significant after adjustment for weight loss in the LME models. **b** Many of the associations shown in **a** were consistent, even after adjusting for weight loss in the LME models. The yellow points represent the associations for which significance was lost after adjustment for weight loss. **c–i** Scatterplots for selected significant associations based on the LME models in **a**. Log-transformed relative abundances of gut bacterial families were corrected for participant-specific offsets using the regression intercepts. The regression slopes for each weight loss quartile has been shown for associations that were significantly influenced by the degree of weight loss (refer to legend) (raw LME model plots without individual-specific abundance correction are shown in Figure S7). **j** Correlation network between bacterial families and anthropometric measurements, clinical markers and food and energy intake based on the data from **a**. The network includes bacterial families with at least one significant association to any of the aforementioned parameters and other families that show significant correlation across LME model coefficients (rows of the heatmap in **a**) with any of those families (Spearman correlation *q* < 0.05, edge thickness proportional to rho). Edges between families and the parameters are included if the absolute effect size (estimated by the LME) exceeded 0.075 (significant associations are indicated by stronger edges). Additional file 7: Source data 6.

Even though we did not observe significant longitudinal changes in gut microbial abundances between intervention or weight loss groups, our study was uniquely powered to identify changes in gut microbiota associated with anthropometric, dietary, clinical, and metabolic biomarkers of obesity and early-stage metabolic syndrome when assessed irrespectively of intervention type. To leverage both the longitudinal sampling and the large number of participants, we subjected gut bacterial families to analysis with linear mixed effect (LME) models [38], which can reveal linear co-variation across timepoints that is consistent across study participants (Fig. 6a, see also Additional file 1: Figure S7 and Methods). Additionally, we also constructed LME models with the weight loss quartiles as a modifying effect, which revealed a subset of associations between bacterial families and clinical biomarkers to be of different strength across weight loss groups (Fig. 6a,b). This analysis showed *Akkermansiaceae*, the only member of the *Verrucomicrobia* phylum found in the human gut, to be inversely correlated with established biomarkers of metabolic dysfunction, namely insulin, HOMA-IR, and triglyceride concentrations (Fig. 6a,c–d). Our data also showed *Christensenellaceae* to be inversely associated with weight and metabolic indicators of (pre-) diabetes and cardiovascular conditions. Specifically, we found this bacterial family to be anti-correlated with BMI, VAT, liver fat, HOMA-IR, and blood concentrations of triglycerides, and cholesterol (Fig. 6a, e–g). *Marinifilaceae* and *Rikenellaceae*, which positively correlated with *Christensenellaceae*, were also inversely associated with VAT. In contrast to *Akkermansiaceae* and *Christensenellaceae*, we observed the abundance of *Tannerellaceae *to be positively associated with HOMA-IR (Fig. 6a, h). Beyond biomarkers of lipid and sugar metabolism, our analyses also uncovered interesting covariation between gut microbial families (especially *Peptostreptococcaceae*, but also *Christensenellaceae* and *Marinifilaceae*) and metabolic indicators of liver damage, i.e., ALT and AST, which are biomarkers of non-alcoholic fatty liver disease (NAFLD).

We also included (self-reported) dietary intake into the LME model analysis. Both *Tannerellaceae* and *Coriobacteriaceae* were significantly inversely correlated with intake of fiber and vegetables (Fig. 6a, h–i). On the other hand, we detected a negative association between *Christensenellaceae* (and to a lesser extent, *Akkermansiaceae*) and processed meat and energy intake (Fig. 6a, g).

To investigate the similarity in association profiles between bacterial families and metabolic biomarkers, anthropometric measures, and food groups, we constructed a correlation network across bacterial families based on the LME model coefficients (Fig. 6j). Focusing on families with at least one significant association, we detected three distinct clusters, two of which were anti-correlated: One cluster, comprising both *Akkermansiaceae* and *Christensenellaceae* among other gut bacterial families, showed inverse correlations with biomarkers of metabolic dysfunction and processed meat and total energy intake. Their co-variation profiles were strongly anti-correlated to those of another small cluster, comprising *Coriobacteriaceae* and *Tannerellacae*, which were associated with lower vegetable and fiber intake, and higher levels of HOMA-IR and other biomarkers of metabolic dysfunction. The two anti-correlated clusters in this network thus revealed a broader grouping of gut microbes by their association profiles with metabolic dysfunction, suggesting that broader shifts in community composition might be associated with more pronounced changes in metabolic dysfunction or its reversal by targeted dietary interventions.

The majority of the described associations between bacteria, metabolic biomarkers, and questionnaire-derived dietary items were not differential depending on weight loss over time (Fig. 6b). Nevertheless, a few associations showed heterogeneity across weight loss quartiles in LME models, with stronger correlations between *Christensenellaceae* and both HOMA-IR and energy intake, and between *Tannerellaceae* and fiber intake, among individuals in the highest weight loss quartile (Fig. 6f–g, i, see also Additional file 1: Figure S7 and “Methods”).

## Discussion

Many studies showing intriguing plasticity in gut microbiome composition in response to dietary changes [47–49] have raised the question of whether intestinal microbiota mediate some of the physiological effects of such interventions. In particular, understanding whether the health benefits of weight loss achieved through calorie restriction are linked to changes in the gut microbiome and its metabolites would open up possibilities for optimizing dietary interventions for intestinal microbiome modulation. Thus, we conducted the first study to longitudinally evaluate gut microbiome composition and metabolite changes following calorie restriction among 147 overweight or obese adults (non-smokers free of any gastrointestinal or chronic diseases) over 1 year in a randomized controlled dietary intervention trial. Notwithstanding significant improvements in body composition and several metabolic parameters, the type of calorie restriction, or the amount of weight lost were not accompanied by substantial and consistent shifts in gut microbiome composition or the abundance of individual bacterial taxa.

We did not observe significant changes in circulating microbial-related metabolites, such as bile acids or SCFAs either, with the exception of choline, which could be of dietary or as it has recently been shown, of microbial origin [50]. The levels of choline decreased with weight loss, consistent with previous reports associating this with improvements in insulin sensitivity [51] and a more favorable cardio-metabolic risk factor pattern in general [52]. Our data overall support the interpretation that the decrease in choline and other observed metabolic improvements upon weight loss were not dependent on the gut microbiome. By contrast, the only strong predictors of post-intervention microbial abundances in our study were their baseline abundances suggesting that changes in gut microbiome composition over time are strongly constrained by an individual’s unique microbiome configuration [53]. Taken together, these results suggest that weight loss (i.e., ~7.5–20% in the highest quartile), achieved by calorie restriction is neither associated with substantial short-term nor persistent long-term changes in gut microbiome composition or microbial metabolites in circulation and that metabolic improvements were mainly a consequence of the favorable changes in body composition (decreases in visceral fat and liver fat), rather than in the gut microbiome. This result on gut microbiome stability is in line with two recent randomized trials that have also reported only minimal effects on gut microbiome composition despite significant weight loss [54, 55].

While dietary intervention was not accompanied by significant changes in the gut microbiome over time, we found microbiome composition at baseline to be moderately predictive of weight loss suggesting that higher *Dorea* abundance may be associated with difficulty in losing excess weight through calorie restriction. This finding adds to previous reports that baseline gut microbiota composition may influence response to dietary weight loss interventions [13, 56]. *Dorea* belongs to the *Clostridia* class within the *Firmicutes* phylum and a higher abundance of this phylum has also been associated with greater energy harvest and obesity [57]. Moreover, most bacteria previously suggested to be predictive of weight loss belong to the class *Clostridia* [56]. However, failed validation of previously proposed baseline microbiome biomarkers in our data and the fact that the *Dorea* association with weight loss did not reach statistical significance in an external data set, despite a similar effect size, highlights the need for additional large-scale dietary intervention studies to resolve these discrepancies.

While our study was likely sufficiently powered (*N* = 147) and entailed a longitudinal component which helped to partially adjust for individual-specific microbiome composition, there are a few caveats to our findings. We cannot completely rule out potential local effects in the intestine (undetectable in plasma or fecal samples) or changes in gut microbiome function or activity that remain undetected in our study. Functional and strain-level analyses would require shotgun metagenomic or metatranscriptomic approaches, and the lack of such data is a limitation of our study. This also precluded an analysis of bacterial toxin genes, such as *tcdA* and *tcdB* encoded in the *Clostridioides difficile* genome, the products of which have recently been reported to affect the response to very low caloric diets among morbidly obese patients [58]. Future studies will have to resolve whether relevant alterations in gut microbial functions can occur without significant compositional changes, complementing findings of gut microbiome plasticity being often higher at the taxonomic than the functional level [39]. Finally, we acknowledge that fasting concentrations of metabolites, as used in our study, may not always best reflect metabolic dysfunction, and particularly systemic bile acid concentrations may be more informative in the postprandial state [59].

While our results are not necessarily in agreement with previous reports [13, 60], it should be noted that many previous weight loss interventions involved much more drastic reductions in calorie intake or stronger manipulations of fiber and macronutrient proportions [13, 61]. These studies mostly found abundance changes in specific gut bacterial taxa in association with specific food groups or macronutrients, particularly fiber [10, 62]. In contrast to these studies, throughout our intervention, participants consumed an “everyday diet” based on healthy eating guidelines. Fiber consumption remained relatively stable at a moderate level, while the most significant change of macronutrient proportions occurred through reduction of calories from fat (Figure S8). These modest changes in dietary composition within the limits of healthy dietary intake guidelines nevertheless resulted in substantial weight loss (~5% on average and ~10.7% in the highest quartile), similar to what other trials have achieved [10, 13], and may have contributed to the high retention rates observed throughout the intervention suggesting much better applicability for disease prevention than some previously reported extreme interventions [47, 58]. At the same time, our findings suggest that a balanced dietary composition during weight loss may help to avoid potential detrimental effects to the microbiome that have been observed as a consequence of restrictive approaches for energy reduction [13, 58].

In contrast to several recent animal experiments and first smaller human trials [63], our study does not point to differential effects of ICR vs. CCR on the microbiome, despite a differential increase in *Lactobacillales* with ICR after 12 weeks. Previous studies reported the genus *Lactobacillus* (order *Lactobacillales*) to increase with ICR in mice [18, 19] and among obese humans after a low-fat dietary intervention [64] or successful weight loss after Roux-en-Y Gastric Bypass [65]. However, considering that the increase in *Lactobacillales* in our study was associated with concurrent decreases in processed meat intake during ICR, we cannot rule out that a reduction in red meat intake rather than ICR may have led to this increase. Such an association would also be consistent with previous studies investigating dietary protein in animal models [66–68]. While adherence to ICR was high in our study, especially during the 12-week intervention phase [22], we acknowledge that the 5:2 diet is a mild form of ICR and that further studies on other types of ICR in relation to the microbiome are needed. However, recent meta-analyses of randomized studies do not suggest stronger effects of ICR vs. CCR diets on established metabolic biomarkers, which may speak against differential effects of ICR on the microbiome [69–71].

In a pooled analysis of all study groups, we found that many gut microbial families co-varied significantly and consistently across participants with host metabolic parameters, forming a network with emerging modules of intercorrelated taxa. In particular, our findings from robust LME models leveraging repeated measurements over time on significant associations of *Christensenellaceae*, *Akkermansiaceae*, *Marinifilaceae*, and *Tannerellaceae* with HOMA-IR (and triglycerides for the former two) underline the potential importance of these bacteria for host metabolic status. Consistent with our observation, previous cross-sectional studies have associated a healthy lipid profile and normal glucose homeostasis with increased abundance of the genus *Akkermansia* [72, 73]. Intriguingly, oral supplementation of *Akkermansia muciniphila* was shown to alleviate insulinemia and to lower the plasma concentrations of cholesterol, inflammatory biomarkers, and markers of liver dysfunction among overweight/obese insulin-resistant adults [74]. Also the *Christensenellaceae* family was previously associated with an overall healthy metabolic status [75, 76] and had been shown to be more abundant among individuals with low BMI in cross-sectional twin studies [77]. Our results complement the earlier studies, as they show that *Christensenellaceae* fluctuates over time around individual-specific levels and inversely co-varies with anthropometric and metabolic biomarkers of obesity and diabetes in a consistent manner across individuals. The significant inverse association of *Peptostreptococcaceae* with both ALT and AST, and to a lesser extent with GGT and CRP, is interesting in light of the discrepant reports on the putative role of this intestinal bacterial family in liver disease (sometimes found to increase, sometimes to decrease, with liver dysfunction) [78, 79]. Our results rather support a potentially disease-protective role. Finally, *Marinifilaceae* have recently been shown to be enriched in adipose tissue samples of obese people without diabetes compared to obese people with type 2 diabetes [80], which is somewhat in line with the inverse association between *Marinifilaceae* and VAT in our study.

As opposed to the above-described network of bacteria related to a favorable pattern of metabolic biomarkers, we also observed an anti-correlated profile characterized by co-variation of *Tannerellaceae* and *Coriobacteriaceae*. To our knowledge, *Tannerellaceae*, which were negatively associated with vegetable and fiber intake but positively with HOMA-IR, have not previously been associated with obesity or diabetes in human studies. In mice, however, the abundance of *Tannerellaceae* was found to increase upon a high-fat diet intervention with concomitant elevations in plasma glucose and lipid concentrations [81]. *Tannerellaceae* were positively correlated with *Coriobacteriaceae* that also showed inverse associations with vegetable and fiber intake. *Collinsella*, the only intestinal genus in the latter bacterial family, has been reported to be enriched among individuals with low fiber intake and to be inversely associated with a Mediterranean dietary pattern [82, 83].

We found a few cases of heterogeneity across quartiles of weight loss in the associations between bacteria, metabolic biomarkers, and food intakes in integrated LME analyses of repeated measurements. However, while our analysis of intervention outcomes suggests that they cannot be easily manipulated by calorie restriction alone, their associations with food components, such as vegetables or processed meat, highlight the promise of future dietary intervention studies; based on our post hoc observations, such studies could attempt to specifically modulate these bacterial families while monitoring concomitant metabolic changes. Together with randomized controlled trials of bacterial supplementation [74], these could help to untangle causality and to achieve weight loss and its associated metabolic benefits not only through direct effects on the host, but also through synergistic modulation of the gut microbiota and its metabolic products.

## Conclusions

In conclusion, our findings suggest that moderate ICR or CCR interventions as well as an overall moderate weight loss induced by calorie restriction (irrespective of which form) may not be associated with significant changes in the gut microbiome of overweight and obese adults, notwithstanding observed metabolic improvements. Nevertheless, baseline microbiome composition, in particular *Dorea*, was moderately predictive of intervention outcome in our study, but further work is needed to independently validate this in comparison to other proposed predictors of weight loss. Despite the lack of consistent intervention effects on microbiome composition, LME model analysis, which leveraged data across all time points and participants, while also accounting for weight loss, revealed several significant and plausible associations of gut bacteria with anthropometric measurements, body composition indices, and biomarkers of metabolic health.

## Supplementary Information

**Additional file 1: Figure S1.** Identification of possibly swapped samples. In the figure, samples are displayed as subject id and time point. The affected samples are 700119_3 in the top panel, 700053_T3 in the middle panel, 700085_T0 and 700085_T2 in the bottom panel. **Figure S2.** NMDS plots of Bray-Curtis dissimilarity matrices did not reveal distinct clustering patterns according to intervention groups at weeks (a) 24 and (b) 50. **Figure S3.** *Prevotella* and *Bacteroides* relative abundance of all 16S amplicon samples (a), and histogram of the distribution of the *Prevotella-to-Bacteroides* ratio (b). Bar Plot showing the distribution of the P/B ratio per intervention group (c), time point (d) and combination of intervention group and time point (e). Correlation between weight loss and P/B ratio pre-intervention (f), post-intervention (g) and changes in P/B ratio (post- minus pre-intervention) (h). There is a nominal association in panel c which disappears with multiple testing correction. **Figure S4.** Body weight and energy, fiber, macronutrients, fruits, vegetables and processed meat consumption across timepoints according to intervention groups. **Figure S5.** Non-metric multidimensional scaling (NMDS) plots of Bray-Curtis dissimilarity distances between samples across all timepoints according to (a) intervention groups and (b) weight loss quartiles. **Figure S6.** Association of baseline *Dorea* abundance and gut microbiota plasticity (baseline to week 12) with long-term weight loss i.e. week 24 and week 50. **Figure S7.** LME plots for all significant associations between bacterial families and anthropometric measures (see Figure 6a in the main text). **Figure S8.** Body weight and intakes energy, fiber, macronutrients, fruits, vegetables and processed meat across timepoints according to weight loss quartiles.

**Additional file 2: Table S1.** Further baseline characteristics of participants according to intervention group. **Table S2.** Further baseline characteristics of participants according to weight loss quartiles. **Table S3.** Diversity indices across timepoints according to intervention groups. **Table S4.** Changes in bacteria (order) abundance across time points according to intervention group. **Table S5.** Changes in bacteria (class) abundance across time points according to intervention group. **Table S6.** Changes in anthropometric measurements, body composition, clinical markers and blood-based biomarkers by weight loss quartiles after 24 and 50 weeks. **Table S7.** Diversity indices across timepoints according to weight loss quartiles. **Table S8.** Intervention effects on plasma bile acids concentrations (nmol/L) after 12 weeks according to weight loss quartiles. **Table S9.** Changes in the concentrations of acylcarnitines and amino acids according to weight loss quartiles after 12, 24 and 50 weeks. **Table S10.** Intervention effects on plasma TMAO, betaine and choline concentrations (μmol/L) across timepoints according to weight lossquartiles. **Table S11.** Comparison of bacteria (phylum) relative abundances across timepoints according to intervention groups. **Table S12.** Comparison of bacteria (class) relative abundances across timepoints according to intervention groups. **Table S13.** Comparison of bacteria (order) relative abundances across timepoints according to intervention groups. **Table S14.** Comparison of bacteria (family) relative abundances across timepoints according to intervention groups. **Table S15.** Comparison of bacteria (genus) relative abundances across timepoints according to intervention groups. **Table S16.** Comparison of within-group bacteria (phylum) relative abundances at each timepoint relative to baseline. **Table S17.** Comparison of within-group bacteria (class) relative abundances at each timepoint relative to baseline. **Table S18.** Comparison of within-group bacteria (order) relative abundances at each timepoint relative to baseline. **Table S19.** Comparison of within-group bacteria (family) relative abundances at each timepoint relative to baseline. **Table S20.** Comparison of within-group bacteria (genus) relative abundances at each timepoint relative to baseline. **Table S21.** Study, sample, experiment and run accession numbers of all 16S data generated in the HELENA Trial.

**Additional file 3. Source data for Figure 2.**

**Additional file 4. Source data for Figure 3.**

**Additional file 5. Source data for Figure 4.**

**Additional file 6. Source data for Figure 5.**

**Additional file 7. Source data for Figure 6.**

## Data Availability

The 16S rRNA amplicon sequencing data from this study are available from the European Nucleotide Archive (ENA) database: accession number PRJEB40697 (https://www.ebi.ac.uk/ena/browser/view/PRJEB40697) (see also Additional file 2: Table S21). Changes in the microbiome relative abundance at the phylum, family, and genus taxonomic levels according to intervention groups are also available on Zenodo at (10.5281/zenodo.4287277) [84]. Information on the concentrations of bile acids, acylcarnitines, amino acids, and choline derivatives at baseline, as well as the changes in the concentrations of these metabolites across timepoints according to both intervention group and weight loss quartiles, is publicly available on Zenodo (10.5281/zenodo.4287277) [84]. Source data for all figures shown in this paper have been provided. All other data is available from the corresponding authors on request, on the basis of a data transfer agreement, which is required according to the informed consent statement used for the study. The 16S rRNA amplicon sequencing data from this study are available from the European Nucleotide Archive (ENA) database: accession number PRJEB40697 (https://www.ebi.ac.uk/ena/browser/view/PRJEB40697) [85] (see also Additional file 2: Table S21). All other data is available from the corresponding authors on request, on the basis of a data transfer agreement, which is required according to the informed consent statement used for the study.

## Abbreviations

ALT: Alanine aminotransferase
AST: Aspartate aminotransferase
AUROC: Area under the receiver operating characteristic curve
BMI: Body mass index
BP: Blood pressure
CCR: Continuous calorie restriction
CRP: C-reactive protein
CTR: Control group
CVD: Cardiovascular disease
DNA: Deoxyribonucleic acid
GGT: Gamma-glutamyl transferase
HDL: High-density lipoprotein
ICR: Intermittent calorie restriction
IFN-γ: Interferon gamma
IGF-1: Insulin-like growth factor 1
IL-6: Interleukin 6
IL-8: Interleukin 8
LC: Liquid chromatography
LME: Linear mixed effect
MS: Mass spectrometry
NMDS: Non-metric multidimensional scaling
PERMANOVA: Permutational analysis of variance
RCT: Randomized controlled trial
rRNA: Ribosomal ribonucleic acid
SAT: Subcutaneous adipose tissue
TNF-α: Tumor necrosis factor-α
VAT: Visceral adipose tissue
