# Supplementary material for: Calorie restriction improves metabolic state independently of gut microbiome composition: a randomized dietary intervention trial
Source: Genome Med. 2022 Mar 14;14:30. doi: 10.1186/s13073-022-01030-0 (PMC8919571; doi:10.1186/s13073-022-01030-0)
Supplement: Supplementary file 1 — Additional file 1: Figure S1. Identification of possibly swapped samples. In the figure, samples are displayed as subject id and time point. The affected samples are 700119_3 in the top panel, 700053_T3 in the middle panel, 700085_T0 and 700085_T2 in the bottom panel. Figure S2. NMDS plots of Bray-Curtis dissimilarity matrices did not reveal distinct clustering patterns according to intervention groups at weeks (a) 24 and (b) 50. Figure S3. Prevotella and Bacteroides relative abundance of all 16S amplicon samples (a), and histogram of the distribution of the Prevotella-to-Bacteroides ratio (b). Bar Plot showing the distribution of the P/B ratio per intervention group (c), time point (d) and combination of intervention group and time point (e). Correlation between weight loss and P/B ratio pre-intervention (f), post-intervention (g) and changes in P/B ratio (post- minus pre-intervention) (h). There is a nominal association in panel c which disappears with multiple testing correction. Figure S4. Body weight and energy, fiber, macronutrients, fruits, vegetables and processed meat consumption across timepoints according to intervention groups. Figure S5. Non-metric multidimensional scaling (NMDS) plots of Bray-Curtis dissimilarity distances between samples across all timepoints according to (a) intervention groups and (b) weight loss quartiles. Figure S6. Association of baseline Dorea abundance and gut microbiota plasticity (baseline to week 12) with long-term weight loss i.e. week 24 and week 50. Figure S7. LME plots for all significant associations between bacterial families and anthropometric measures (see Figure 6a in the main text). Figure S8. Body weight and intakes energy, fiber, macronutrients, fruits, vegetables and processed meat across timepoints according to weight loss quartiles. [file 13073_2022_1030_MOESM1_ESM.docx]

# Supplementary Figures


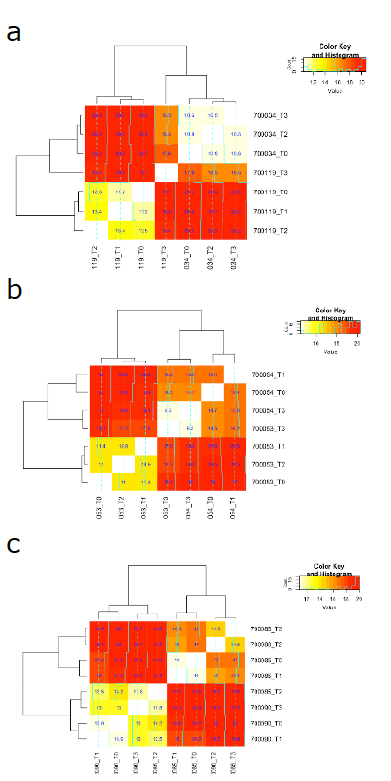


Figure S1. Identification and correction of possibly swapped samples.


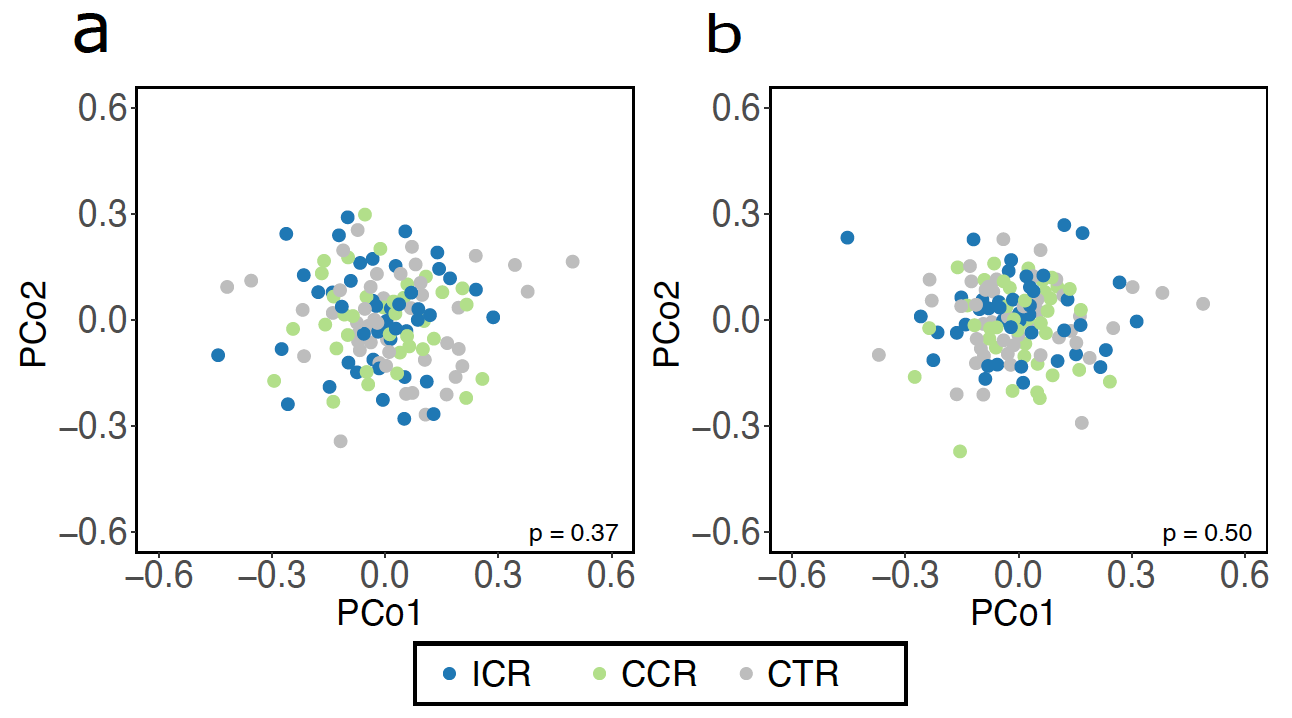


Figure S2. NMDS plots of Bray-Curtis dissimilarity matrices did not reveal distinct clustering patterns according to intervention groups at weeks (a) 24 and (b) 50.

Each point represents the microbial community of a sample, and the colour indicates the intervention group to which it belongs. PERMANOVA did not indicate a statistically significant difference between the group.


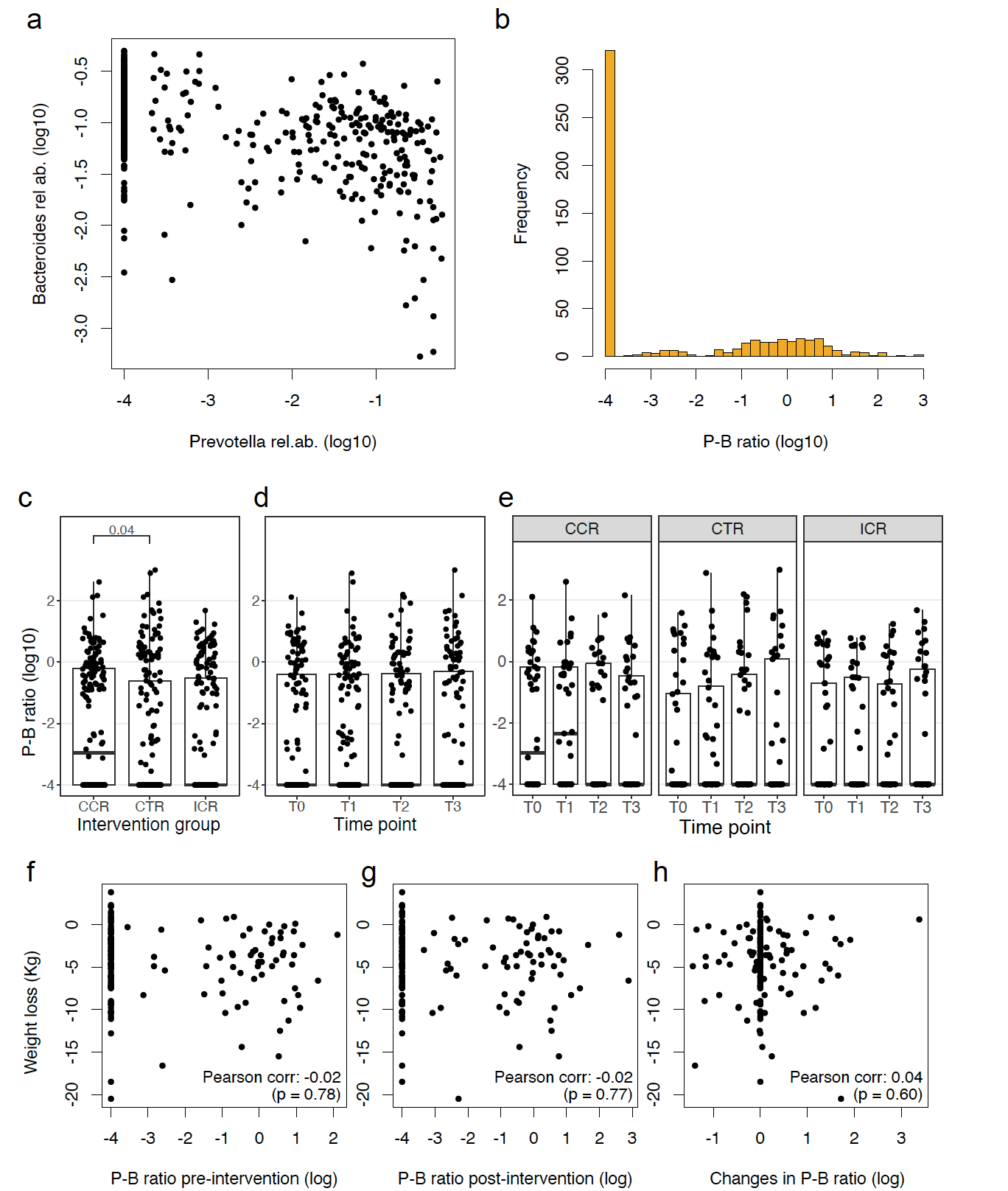


**Figure S3.** ***Prevotella* and *Bacteroides* relative abundance of all 16S amplicon samples (a), and histogram of the distribution of the Prevotella-to-Bacteroides ratio (b). Bar Plot showing the distribution of the P-B ratio per intervention group (c), time point (d) and combination of intervention group and time point (e). Correlation between weight loss and P-B ratio pre-intervention (f), post-intervention (g) and changes in P-B ratio (post- minus pre-intervention) (h). There is a nominal association in panel c which disappears with multiple testing correction.**


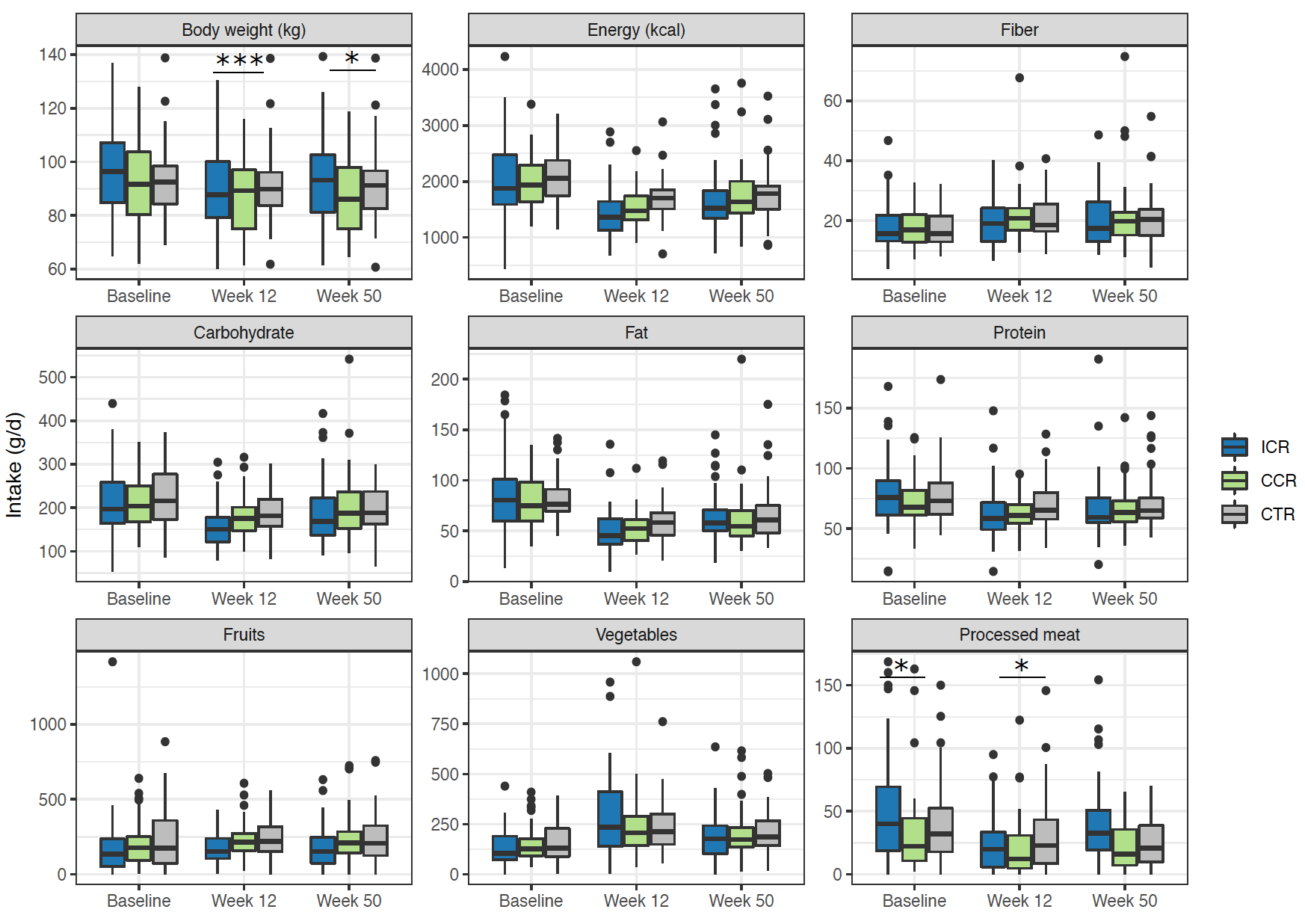


Figure S4. Body weight and energy, fibre, macronutrients, fruits, vegetables and processed meat consumption across timepoints according to intervention groups. All boxplots show the interquartile ranges (IQRs) as boxes, with the median as a black horizontal line and the whiskers extending up to the most extreme points within 1.5-fold IQR.


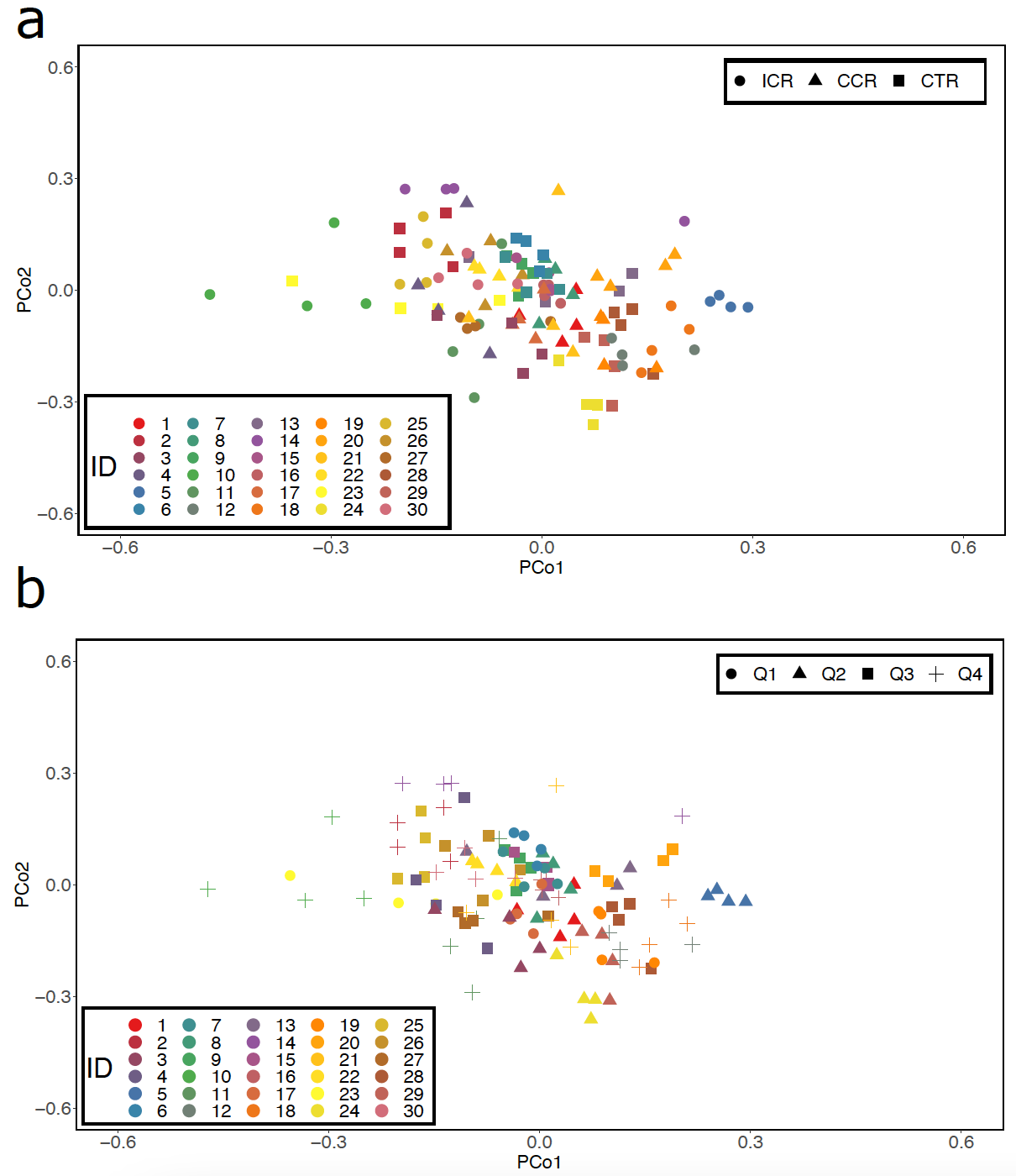


Figure S5. Non-metric multidimensional scaling (NMDS) plots of Bray-Curtis dissimilarity distances between samples across all timepoints according to (a) intervention groups and (b) weight loss quartiles.

NMDS plots did not reveal clustering according to intervention groups i.e. ICR vs. CCR vs Control as depicted in (a) nor according to weight loss quartiles as depicted in (b). Instead, there was strong clustering according to participants, indicating low intraindividual variability over time. Each point represents the microbial community of a sample, and the colour indicates the intervention group to which it belongs.


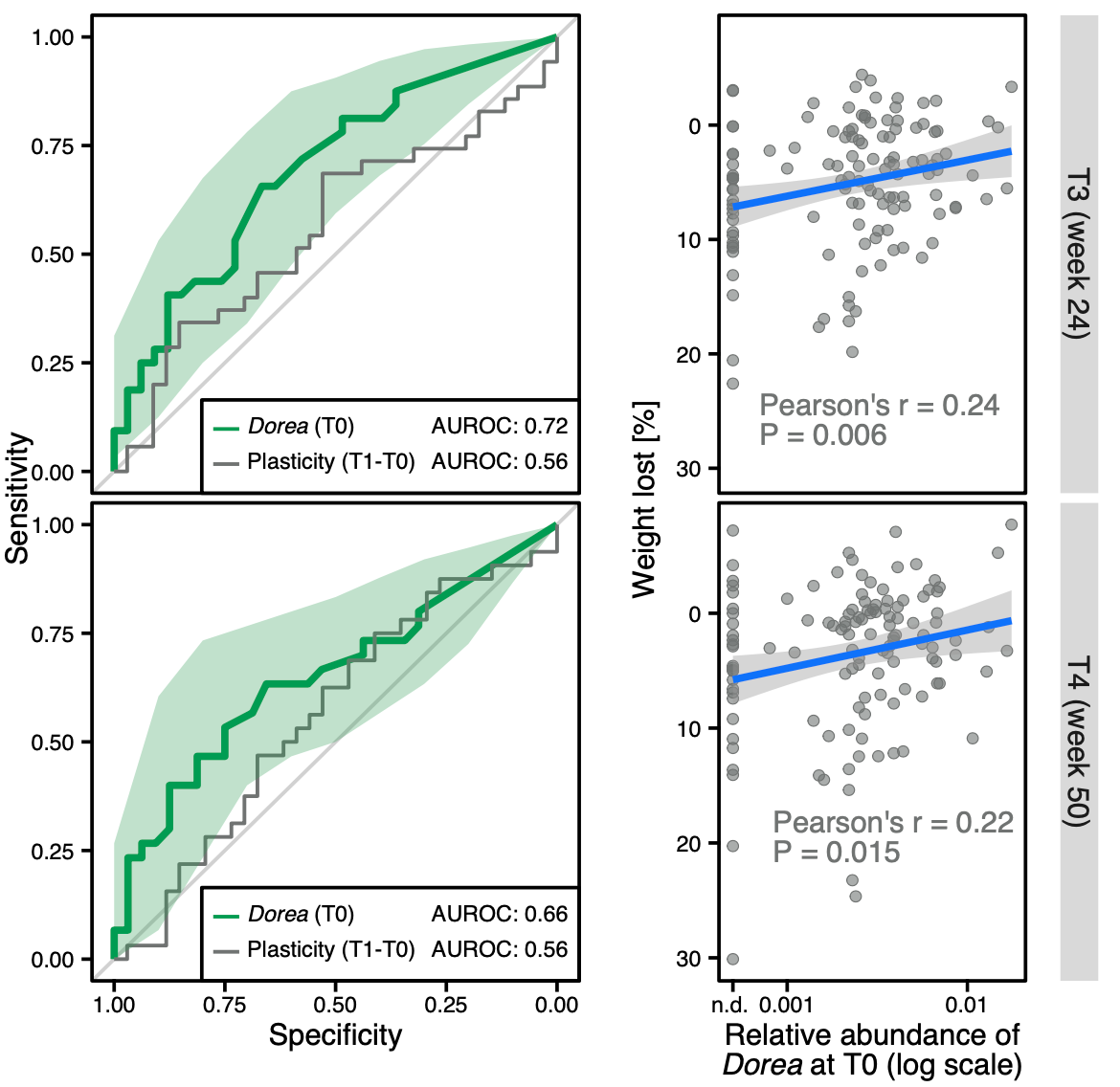


**Figure S6. Association of baseline *Dorea* abundance and gut microbiota plasticity (baseline to week 12) with long-term weight loss i.e. week 24 and week 50.**

Figure S7. Raw LME plots for all significant associations between bacterial families and anthropometric measures (see Figure 6a in the main text).


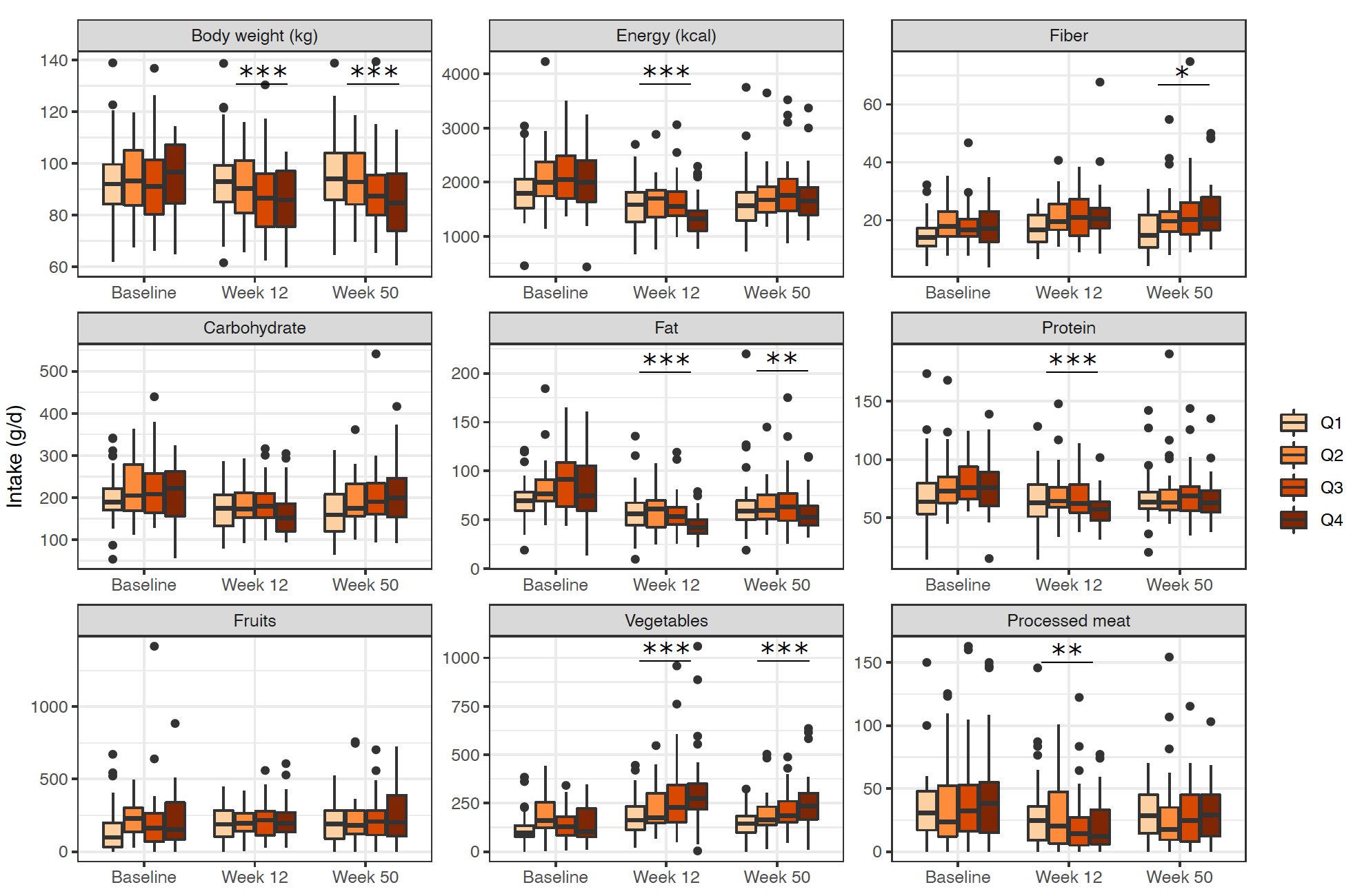


Figure S8. Body weight and intakes of energy, fibre, macronutrients, fruits, vegetables and processed meat across timepoints according to weight loss quartiles. All boxplots show the interquartile ranges (IQRs) as boxes, with the median as a black horizontal line and the whiskers extending up to the most extreme points within 1.5-fold IQR.
